# Vector-Borne Bacterial Plant Pathogens: Interactions with Hemipteran Insects and Plants

**DOI:** 10.3389/fpls.2016.01163

**Published:** 2016-08-09

**Authors:** Laura M. Perilla-Henao, Clare L. Casteel

**Affiliations:** Department of Plant Pathology, University of California at Davis, Davis, CAUSA

**Keywords:** vector-borne bacteria, vascular bacteria, phloem, xylem, plant–insect interactions, plant–microbe interactions, leafhoppers, psyllids

## Abstract

Hemipteran insects are devastating pests of crops due to their wide host range, rapid reproduction, and ability to transmit numerous plant-infecting pathogens as vectors. While the field of plant–virus–vector interactions has flourished in recent years, plant–bacteria–vector interactions remain poorly understood. Leafhoppers and psyllids are by far the most important vectors of bacterial pathogens, yet there are still significant gaps in our understanding of their feeding behavior, salivary secretions, and plant responses as compared to important viral vectors, such as whiteflies and aphids. Even with an incomplete understanding of plant–bacteria–vector interactions, some common themes have emerged: (1) all known vector-borne bacteria share the ability to propagate in the plant and insect host; (2) particular hemipteran families appear to be incapable of transmitting vector-borne bacteria; (3) all known vector-borne bacteria have highly reduced genomes and coding capacity, resulting in host-dependence; and (4) vector-borne bacteria encode proteins that are essential for colonization of specific hosts, though only a few types of proteins have been investigated. Here, we review the current knowledge on important vector-borne bacterial pathogens, including *Xylella fastidiosa*, *Spiroplasma* spp., *Liberibacter* spp., and ‘*Candidatus* Phytoplasma spp.’. We then highlight recent approaches used in the study of vector-borne bacteria. Finally, we discuss the application of this knowledge for control and future directions that will need to be addressed in the field of vector–plant–bacteria interactions.

## Introduction

The plant vascular system is a rich source of nutrients and represents a transport pathway for colonizers. It consists of phloem and xylem tissues, two different host environments for plant pathogens. Phloem tissue consists of companion cells, providing metabolic and regulatory components to the phloem sap, and sieve elements, forming a long distance transport system throughout the plant (Lucas, 2006; Will et al., 2013). Because this specialized transport system offers access to a rich source of carbohydrates, proteins, and amino acids, numerous viral and bacterial microbes colonize the phloem specifically (Bove and Garnier, 2003; Lough and Lucas, 2006). In contrast, the xylem vessels mainly transport water and contain lower nutrient levels in comparison to the phloem (Bae et al., 2015). Despite the low nutrient content of xylem, plant pathogens have also been identified that can colonize the xylem (Purcell and Hopkins, 1996; Bae et al., 2015).

In addition to viral and bacterial microorganisms, macro organisms also rely on the plant vascular system for their primary nutrient source. These include hemipteran pests, such as whiteflies, aphids, psyllids, and leafhoppers. Specialized mouthparts, known as stylets, allow hemipterans to penetrate the plant’s epidermal tissues and reach their preferred tissue. Some hemipterans feed from the mesophyll and vascular system, while others only probe the mesophyll and feed exclusively from the phloem or xylem. As a result of this specialized feeding, hemipterans interact with microbes colonizing the plant vascular system and can serve as vectors. A vector is the specific organism that transmits a pathogen (Purcell, 1982) and hemipteran insects are by far the most important vectors of plant-infecting pathogens (Nault and Ammar, 1989; Orlovskis et al., 2015).

While the interactions between plant–pathogenic viruses and their hemipteran vectors have been studied in depth, far less is known about the interactions between plant–infecting bacteria and their hemipteran vectors (Ng and Falk, 2006; Hogenhout et al., 2008a; Walling, 2008; Ammar et al., 2011; Blanc et al., 2011, 2014; Gray et al., 2014; Gilbertson et al., 2015; Whitfield et al., 2015). In recent decades, vector-borne bacteria have caused some of the most devastating plant diseases in perennial and annual crops. For example, in North America ‘*Candidatus* Liberibacter asiaticus,’ the causative agent of citrus greening, has rapidly spread across several regions of the world. Citrus greening continues to cost growers over $4 billion each year and has resulted in the loss of 1000s of jobs (Gottwald, 2010). Here, we review the current mechanistic knowledge of interactions shared among vector-borne bacteria, hemipteran vectors, and host plants. As most vector-borne bacteria cannot be cultured and are difficult to study in the lab, we then highlight current approaches used to study these tri-partite systems. Finally, we discuss application of recent knowledge for control and propose future directions for research on vector-borne bacteria and their hemipteran vectors.

## Redefining the Relationships Vector-Borne Bacteria Share With Hemipteran Insects

Early studies of plant pathogens used microscopy, serological testing, and host inoculation to determine the etiological agents of diseases. While insect transmission of plant viruses was first described in 1920, insect transmission of plant bacteria was not reported until 1967 (Purcell, 1982). Because of the historic precedence of research on vector-borne viruses, concepts and terminology from virus research were applied to the study of vector-borne bacteria. In spite of this methodological connection, the actual similarities between viruses and bacteria as vector-transmitted plant pathogens may be quite limited. Here, we briefly define the common terminology found in the literature for describing pathogen–vector interactions and highlight terms that are useful for vector-borne bacteria specifically.

### Persistence: Non-Persistent, Semi-persistent, or Persistent

The transmission process of vector-borne viruses is categorized by two features: (1) the time period required by the vector for acquisition of the virus and inoculation of the virus, and (2) the retention time of viral particles in the vector (Ng and Falk, 2006). Based on these features, virus-vector relationships can be categorized as non-persistent, semi-persistent, or persistent. For non-persistent viruses, transmission can occur within minutes of acquiring the viral particles (virions) and particles are retained in the stylet or in the alimentary canal of the insect (Ng and Falk, 2006; Uzest et al., 2007; Whitfield et al., 2015). Viral particles can be lost quickly in this transmission mode and multiple encounters with infected plants are required for vectors to remain viruliferous (Ng and Falk, 2006). Semi-persistent retention of virions can last for days and retention sites are found in the alimentary canal or gut lumen of the insect for the majority of these viruses (Chen et al., 2011; Ng and Zhou, 2015). For semi-persistent relationships, feeding for hours to days is required to acquire the virus and if acquisition occurs during vector immature stages, infectivity is lost after each molt. Finally for persistent associations, vectors remain infective until death after a single encounter with an infected plant. Long feeding periods (hours to days) are required for acquisition of persistent viruses by vectors.

Persistence of vector-borne bacteria varies according to plant-tissue specialization. *Xylella fastidiosa*, the only known vector-borne xylem specialist, has a semi-persistent association with its vectors (**Table 1**). Dozens of crops and native plants are hosts for *X. fastidiosa* and a diverse array of vectors transmits the pathogen compared to other species of vector-borne bacteria (Redak et al., 2004). The ability to utilize diverse plant and vector species may be due to *X. fastidiosa*’s semi-persistent relationship with insects. For example, semi-persistent bacteria may be more easily acquired and transmitted by vectors to diverse host species during pre-feeding and host finding behavior. In contrast, all known phloem-limited bacteria appear to establish persistent associations with their respective vectors (**Table 1**). Persistence of phloem specialists may be due to the intracellular relationship they share with plant and insect hosts. However, conclusion about tissue trophisms may be premature, as only one vector-borne xylem specialists is known so far.

**Table 1:** Vector-borne phloem limited plant pathogenic bacteria.

Class Family	Pathogen	Genome size (Mb)	Plant tissue tropism	Plant host	Vectors	Location/ Insect organs	Reference
Gammaproteobacteria *Xanthomonadaceae*	*Xylella fastidiosa*	2.7	Xylem **(a)**	Wide host range	*Homalodisca vitripennis, Graphocephala atropunctata* (+ Others)	Non-Circulative/ Cybarium, foregut	Backus and Morgan, 2011
Mollicutes *Spiroplasmataceae*	*Spiroplasma citri*	1.8	Phloem **(a)**	Citrus	*Circulifer tenellus*	Circulative/	Fletcher et al., 1998
	*Spiroplasma kunkelii*			Corn	*Dalbulus maidis*	Hemolymph bacteriocyte, salivary glands	
Mollicutes *Acholeplasmataceae*	*“Candidatus* Phytoplasma spp.”	0.8	Phloem **(b)**	Wide host range: Asteraceae horticulture crops	*Macrosteles quadrilineatus* (+ Others)	Circulative/ Hemolymph bacteriocyte, salivary glands	Beanland et al., 2000
Alphaproteobacteria *Rhizobiaceae*	“*Candidatus* Liberibacter spp.”	1.2	Phloem **(b)**	Citrus Solanaceae Apiaceae	*Diaphorina citri*, *Bactericera cockerelli, Bactericera trigonica, Trioza apicalis*	Circulative/ Hemolymph bacteriocyte, salivary glands	Ammar et al., 2011

*The bacterial group is routinely culturable **(a)** or non-culturable **(b)**.*

### Location: Circulative or Non-circulative

The interactions plant viruses share with their insect vectors can either be “non-circulative” or “circulative.” In non-circulative interactions, the virus does not enter the insect body as part of the transmission process and the virus particles are retained in the stylet or the foregut region (Ng and Zhou, 2015). Viruses that are transmitted in a circulative manner in contrast, pass beyond the foregut into the insect intestine and enter the body as part of the transmission process. Circulative viruses can be retained for the life of the insect vector (Gray et al., 2014). For vector-borne bacteria both non-circulative and circulative relationships exist among pathogen–vector interactions (**Table 1**); and like persistence, relationships correlate with plant-tissue specialization of the pathogen. For example, the xylem colonizer, *X. fastidiosa*, is non-circulative, while all known phloem colonizers interact in a circulative manner with vectors (**Table 1**).

Differences in pathogen location within vectors may be explained by ancestral origins (Nadarasah and Stavrinides, 2011). In one scenario, bacteria pre-adapted to plant environments may have evolved to use insects as alternative hosts. Alternatively, insect pathogens or symbionts, pre-adapted to thrive in hemipterans, may have found an additional niche in plants (Nadarasah and Stavrinides, 2011). *X. fastidiosa* is most closely related to the genus *Xanthomonas* (**Table 1**). Members of *Xanthomonas* are exclusively plant-associated and commonly plant pathogens. Inability to cross insect membranes may be due to the fact that *X. fastidiosa* has evolved to be restricted to dead cells of the plant (xylem). The ability to cross plant cellular membranes may have been lost from its genetic arsenal over time. Liberibacters also are related to plant pathogens as a member of the family Rhizobiaceae, yet liberibacters have circulative relationships with insect vectors. A more striking phylogenetic observation for liberibacters is that many members of the Rhizobiaceae, have intracellular associations with hosts as pathogens and symbionts (insect and plant hosts; genera *Bradyrhizobium*, *Bartonella*, *Brucella*, and *Afipia*; Jagoueix et al., 1994). This trend may explain the origin of the circulative associations of liberibacters with their vectors. As microbiome projects for hemipterans expand, the relationship among these bacteria and the traits responsible for interactions inside the insect will likely be revealed.

### Replication: Propagative or Non-propagative

Circulative viral pathogens can either circulate through the insect vector’s body without reproducing, in which case they are described as “non-propagative,” or they can circulate and multiply within the insect vector, in which case they are described as “propagative.” In the latter case, the vector serves as an alternative host for the plant pathogen (Nadarasah and Stavrinides, 2011). Typically, the vector acquires the plant pathogen by feeding on infected plants. Once inside the insect body, the virus crosses intestinal barriers, internal organs, and visceral muscles, and can be found throughout the hemolymph (Hogenhout et al., 2008a; Orlovskis et al., 2015). From the hemolymph the virus must spread to the salivary glands before the vector can subsequently transmit the pathogen to a new plant host. Only a few families of vector-borne plant viruses have propagative relationships with vectors. These families include *Rhabdoviridae*, *Reoviridae*, and *Bunyaviridae* (Hogenhout et al., 2008a; Ammar et al., 2009; Whitfield et al., 2015).

All described vector-borne bacteria utilize their insect vectors as alternative hosts, and are thus considered propagative (Bove and Garnier, 2003; Orlovskis et al., 2015). Vector-borne bacteria can propagate extracellularly (between host cells) or intracellularly (within host cells; **Table 1**). For example, the xylem colonizer, *X. fastidiosa*, propagates extracellularly within the vector and is non-circulative. This is in contrast to all vector-borne viruses, which can only be propagative and circulative, as they are all parasites of the cellular replication machinery. It is assumed all phloem colonizers propagate intracellularly within their vectors as they are found in diverse tissues and hemolymph (**Table 1**). However, detailed intracellular propagation of bacteria is not easily studied and the current knowledge may reflect methodological limitations for evaluating bacterial replication in different insect organs and cavities. Specific mechanisms mediating insect recognition, attachment, and multiplication in organs are also not yet clear, and seem to be unique for each bacteria–vector interaction.

## Hemipterans and Their Role as Vectors of Bacterial Plant Pathogens

**FIGURE 1:**
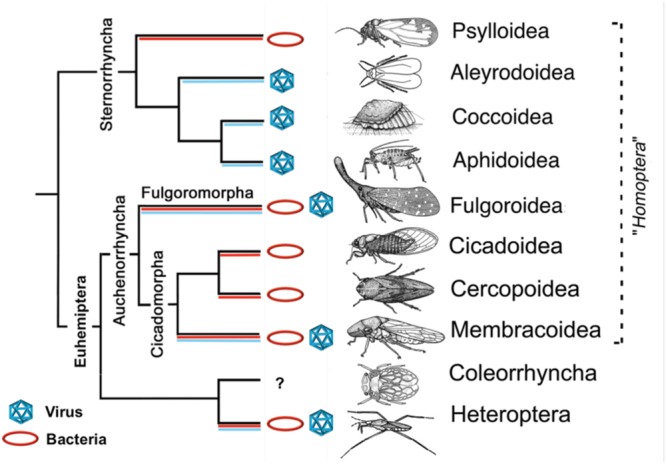
**Hemiptera taxa: reported vectors and groups of plant pathogens.** Specific plant pathogens are listed in Supplementary Table S1. Branches where species have been reported as transmitting virus are labeled in blue, while those transmitting bacteria are labeled in red. Figure modified with permission from Gullan and Cranston (2014).

The ability to serve as a viral and/or bacterial vector appears to vary across hemipteran lineages (**Figure 1**; Supplementary Table S1). Vector-borne bacteria most commonly rely on members of the suborder Auchenorrhyncha for transmission, including leafhoppers (Membracoidea), froghoppers/spittlebugs (Cercopoidea), and planthoppers (Fulgoroidea; **Figure 1**) (Bove and Garnier, 2003). However, several psyllids (Psylloidea) from the subgroup Sternorrhyncha are also important vectors of bacterial plant pathogens (**Figure 1**; Supplementary Table S1). In these groups, transmission has been demonstrated for mesophyll, xylem, and phloem-feeding hemipterans (**Figure 1**; Supplementary Table S1). The efficiency of pathogen transmission, however, depends on the specific insect–plant interaction and on pathogen biology.

Vectors of bacterial plant pathogens and vectors of viral plant pathogens have been reported in multiple superfamilies of the Euhemiptera lineage (Auchenorrhyncha, Coleorrhyncha, and Heteroptera; **Figure 1**). Surprisingly, only a few individual species within these superfamilies have been reported to serve as efficient vectors for both bacterial and viral plant pathogens (Weintraub and Beanland, 2006). One such example is the Beet Leafhopper (*Circulifer tenellus*, Baker), which is a vector for the *Beet curly top virus* ([BCTV], *Geminiviridae*), as well as two different bacterial pathogens (“*Candidatus* Phytoplasma trifolii” and *Spiroplasma citri*; Weintraub and Beanland, 2006).

The suborder Sternorrhyncha contains psyllids, aphids (Aphidoidea), whiteflies (Aleyrodoidea), and scales (Coccoidea), though the latter three are more closely related to one another phylogenetically (Gullan and Cranston, 2014). This is interesting because aphids, whiteflies, and scales have only been reported as vectors of viruses, while psyllids have only been reported as vectors of bacteria (**Figure 1**; Supplementary Table S1). In fact, aphids and whiteflies are the most important vectors of plant viruses, transmitting 46% of all described plant-infecting viruses (Hogenhout et al., 2008a; Gilbertson et al., 2015). Recent work on the Asian citrus psyllid’s (*Diaphorina citri*) viral metagenome found viral sequences from diverse groups of animal viruses but no sequences related to any known viral plant pathogens were reported (Nouri et al., 2015). These results provide further evidence that psyllids may lack the ability to transmit plant viruses, however, additional work in this area is needed.

Our literature review suggests that some hemipteran groups are capable of transmitting bacterial pathogens while other groups are not (**Figure 1**). Despite extensive research into various vector systems, the mechanisms that mediate vector specificity remain largely unknown for all but a few vector-borne phytopathogens (Uzest et al., 2007; Chen et al., 2011; Blanc et al., 2014). Variations in vector specificity among lineages suggest physical, physiological, or temporal constraints on vector-pathogen relationships. Vectors that transmit viruses and bacteria may have fewer constraints, or constraints that are easier for the pathogens to overcome. Differences in insect physiology, immunity, or feeding behavior among groups may mediate some aspects of vector specificity. However, conclusions on differences in insect biology are difficult to make at this point, as the basic biology of many hemipterans remains poorly understood and complete genomes are available for only a few vectors of plant pathogens (Leshkowitz et al., 2006; Ramsey et al., 2007; Legeai et al., 2010; Chen et al., 2015; Upadhyay et al., 2015). Variations in vector specificity may also depend on location and timing of vectors and pathogens. Geographic factors, environmental conditions, and agricultural economics are all dynamic forces that may limit host distribution, insect populations, and plant-pathogen-vector associations. Alternatively, these observations may be the result of a lack of information on the full extent of vector-borne pathogens and their hemipteran vectors, as the current inventory is still likely underrepresented (Malmstrom et al., 2011).

## Vector-Borne Bacteria: Dual Host Interactions

All known vector-borne bacteria share certain biological features, including plant vascular tissue specialization, propagative relationships with vectors, and complete dependence on their hosts. Host dependence is likely a result of genome degradation, where essential biosynthetic pathways from the bacterial ancestor have been lost where the same resources can be obtained from the host environment (Nadarasah and Stavrinides, 2011). The xylem colonizer, *X. fastidiosa*, has the largest genome of the group (**Table 1**). This may be due to the fact that xylem represent an inferior nutrient sources as compared to the phloem. Therefore, more essential biosynthetic pathways in the genome may be required for the bacteria to survive in the nutrient limited xylem. Despite these similarities, the bacterial groups that depend on hemipteran vectors for transmission occur in several different phyla and orders (**Table 1**). Accordingly, many differences exist, including diverse mechanisms for promoting host colonization and dispersal (Orlovskis et al., 2015).

### Xylem-Limited Vector-Borne Bacteria

The only known xylem-limited bacterial pathogen that is also transmitted by hemipteran vectors is *X. fastidiosa. X. fastidiosa* (class Gammaproteobacteria) has a very wide host range, colonizing and causing disease in grapes (Pierce’s disease), citrus (citrus variegated chlorosis), olives (leaf scorch), almonds (leaf scorch), and several other plant species (Chatterjee et al., 2008a; Saponari et al., 2014; Almeida and Nuney, 2015). Because of its economic importance, *X. fastidiosa* was the first bacterial plant pathogen genome to be completely sequenced (Simpson et al., 2000). The genome of *X. fastidiosa* is ~2.7 Mb, half the size of its closest relatives (Xanthomonas group; **Table 1**). *X. fastidiosa* is transmitted in a non-circulative manner by a diverse set of xylem-feeding hemipterans including members from the superfamilies Membracoidea, Cercopoidea, and Cicadoidea (PaiãO et al., 1996; Purcell and Hopkins, 1996; Saponari et al., 2014). Experimental evidence suggests that a wide range of additional xylem feeding hemipterans are potential vectors, however, efficiency of transmission may vary depending on the vector species (Redak et al., 2004). The planthoppers *Homalodisca vitripennis* and *Graphocephala atropunctata* are the two most well studied vectors of *X. fastidiosa*. Knowledge of these two vectors has been used to model the relationship *X. fastidiosa* share with vectors in general (Chatterjee et al., 2008b; Backus and Morgan, 2011; Rapicavoli et al., 2015).

Numerous research tools have been developed to study *X. fastidiosa*, including *in vitro* culture techniques, transformation, and bacterial strain mutants (Killiny et al., 2012; Purcell, 2013; Webster et al., 2014; Rapicavoli et al., 2015). These tools have facilitated the dissection of many genetic components involved in pathogenicity. *X. fastidiosa* colonize and propagate extracellularly in the plant and insect (Chatterjee et al., 2008a), with some overlapping mechanisms. *X. fastidiosa* uses a cell-to-cell signaling sensor (RpfC), which acts as a negative regulator. This signaling system is mediated by diffusible signaling factors (DSF) in order to modulate different aspects of behavior in a population dependent manner (i.e., quorum sensing). DSF is secreted into the extracellular environment, activating motility, biofilm formation, and virulence mechanisms when a threshold concentration is reached outside the cell (Chatterjee et al., 2008a). Aspects of *X. fastidiosa* colonization that are dependent on quorum sensing include the production of toxins, extracellular polysaccharides (EPS), adhesins, and hemaglutinins (**Table 2**) (Chatterjee et al., 2008b; Nascimento et al., 2016). Inside the insect, the bacteria do not invade the epithelial gut, hemolymph, or salivary glands, and are retained in the alimentary canal. Transmission can occur within minutes of acquisition (Killiny et al., 2012). Recently it was determined that lipopolysaccharide (LPS), the outermost layer of sugar polymers surrounding Gram-negative bacteria, is critical for attachment to the vector and subsequent transmission (**Table 2**) (Rapicavoli et al., 2015).

**Table 2:** Reported gene product (or structure) associated with host interaction for vector-borne bacteria.

Vector-borne bacteria	Gene product	Descriptions	Mechanisms	Phenotype	Reference
*Xylella fastidiosa*	RpfC	Signaling sensor	Negative regulator for DSF	Mutants show hyper attachment phenotype in xylem vessels and cybarium of insect vector	Chatterjee et al., 2008b
	FimA, FimF	Type I fimbrial adhesins	Facilitates cell–cell aggregation	–	Chatterjee et al., 2008b
	HxfA, HxfB	Hemaglutinins	Facilitates cell–cell aggregation and cell–surface interactions	HxfA mutants slightly reduced attachment	Chatterjee et al., 2008b
	PglA	Polygalacturonase	–	Mutants lack pathogenicity and systemic movement in plants	Roper et al., 2007
	β-1,4 endoglucanases Xylanases Xylosidases	Cell degrading enzymes	Degradation of plant cell wall components	–	Chatterjee et al., 2008b
	LesA	Lipase/esterase Type II toxin	Phytotoxicity	Phytotoxicity in early plant infection	Nascimento et al., 2016
	*O*-antigen in LPS	*O-*Lipopolysaccharide		Mutants lack full pathogenicity	Rapicavoli et al., 2015
*Spiroplasma kunkelii*	***–***	Pili, extracellular structure	Attachment to insects	–	Ammar et al., 2004
*Spiroplasma citri*	P58	Membrane protein	Attachment to insects	–	Ye et al., 1997
	SARP1	Membrane protein	Attachment to insects	–	Berg et al., 2001
	Spiralin	Membrane protein	Attachment to insects	Mutants have reduced transmission by insects	Gasparich, 2010
	P32	Membrane protein encoded in plasmid (pSci6)	Attachment to insects	Mutants have reduced attachment to insects	Berho et al., 2006
“*Candidatus* Phytoplasma spp.”	SAP11	Sec-exported, NLS signal	Block JA biosynthesis in plants		Sugio et al., 2011
	Amp	Sec-exported, Transmembrane domain	Interacts with insect proteins	Increase vector fecundity	Rashidi et al., 2015
	SAP54/PHYL	Sec-exported	Interaction floral transcription factors	Floral abnormalities as phyllody	MacLean et al., 2014
		NLS signal	Degrades MADS-box proteins		Maejima et al., 2014
	TENGU	Sec-exported	Inhibits auxin-related pathway	Dwarf plants	Minato et al., 2014
	P38	Adhesin domain	Interacts with insect proteins	–	Neriya et al., 2014
	HflB	Protease	Virulence factor	–	Seemüller et al., 2013
	VmpA	Membrane protein	Interaction with insects	–	Renaudin et al., 2015
“*Candidatus* Liberibacter asiaticus”	LasAI	Autotransporter	Unknown	–	Hao et al., 2013
	SC2_gp095	Glutathione peroxidase	Detoxify ROS	–	Jain et al., 2015

In plants, *X. fastidiosa* relies on bacterial multiplication, attachment, and dispersion into neighboring vessels to colonize the xylem (Chatterjee et al., 2008b; Nascimento et al., 2016). Phytotoxicity during early stages of infection is associated with the lipase/esterase effector LesA, a type II secreted enzyme produced abundantly in culture (Nascimento et al., 2016). By degrading plant cell walls, nutrients are acquired and the bacteria are able to disperse throughout the plant (Purcell, 2013; Fatima and Senthil-Kumar, 2015). Degradation of vascular plant tissue requires the combined action of multiple enzymes, such as β-1,4 endoglucanases, xylanases, xylosidases, and polygalacturonases (Purcell, 2013; Fatima and Senthil-Kumar, 2015). *X. fastidiosa* mutants impaired in polygalacturonases enzyme (PglA) production, lack pathogenicity, and systemic movement in the plant (**Table 2**) (Roper et al., 2007). Given the reduced nutritional content of the xylem and the extracellular location of *X. fastidiosa*, many differences may exist for pathogenicity strategies among xylem and phloem colonizers.

### Phloem-Limited Vector-Borne Bacteria

Diverse phylogenetic groups converge in phloem specialization and hemipteran transmission and it is hypothesized that those traits have been acquired independently multiple times over the course of bacteria evolution (Orlovskis et al., 2015). The majority of phytoplasmas (class Mollicutes) and liberibacters (class Alphaproteobacteria) are vector-borne phytopathogens (Bressan, 2014; Fagen et al., 2014a). For other groups, such as spiroplasmas (class Mollicutes), only some species are phytopathogens. Despite this diversity, all known phloem-limited vector-borne bacteria appear to colonize both the insect vector and the plant host intracellularly (Orlovskis et al., 2015). The bacteria cross the gut barrier and circulate in the vector body, eventually reaching the hemolymph, and salivary glands (**Table 1**) (Gasparich, 2010). The journey to the salivary glands requires a latent period, ranging from days to months, before transmission can occur (Thebaud et al., 2009). Here, we will discuss three examples of phylogenetic groups containing vector-borne bacteria: spiroplasmas, phytoplasmas, and liberibacters.

### Spiroplasmas

*Spiroplasma* spp. have a distinctive helical morphology and use pili–like structures to move in a corkscrew-like motion (Ammar et al., 2004). They are classified as Mollicutes as they lack a cell wall. Spiroplasmas share diverse relationships with plant and insect hosts spanning pathogenic, commensal, and mutualistic interactions (Ammar et al., 2004). Most are associated with diverse insect orders, such as Hymenoptera, Coleoptera, Diptera, Lepidoptera, and Hemiptera (Gasparich, 2010). However, there are three phytopathogenic spiroplasmas that are also transmitted by leafhoppers (Cicadellidae): *Spiroplasma citri*, *S. kunkelii*, and *S. phoeniceum* (**Table 1**) (Gasparich, 2010). *S. citri*, the causal agent of citrus stubborn, was the first vector-borne bacteria to be cultured. It was first discovered in 1970 and culture methods were developed shortly after this (Bove and Garnier, 2003). Cultivation of spiroplasmas is not trivial, as it requires complex media enriched with cholesterol and fatty acids. Tools and information derived for spiroplasmas culture methods have served as references for attempts to culture phytoplasmas and liberibacter.

After acquisition, spiroplasmas adhere to receptors in the lumen of the insect midgut, where endocytosis occurs (Fletcher et al., 1998; Gasparich, 2010). Intracellular vesicular transport mediates migration to the hemolymph and exocytosis (Fletcher et al., 1998). Once inside the hemolymph, the bacteria are transported throughout the insect body, eventually reaching the salivary glands after additional intracellular crossings (Fletcher et al., 1998). Currently, the specific insect receptors/factors mediating the journey inside the vector remain unknown for spiroplasmas. However, several potential proteins required for insect attachment have been identified using *S. citri* mutants impaired in insect transmission and with *S. citri* strains that have lost insect attachment properties after multiple *in vitro* cultivations (Ye et al., 1997; Fletcher et al., 1998; Berho et al., 2006; Mutaqin et al., 2011) (**Table 2**).

One of the first approaches developed to study bacterial protein–insect interactions was the use of leafhopper (*C. tenellus*) monolayer cell culture assays with spiroplasmas. In this technique, researchers exposed insect cells (CT1) *in vitro* to *S. citri*. After exposure, electron microscopy (Wayadande and Fletcher, 1998) or immunofluorescence assays (Labroussaa et al., 2010) were used to evaluate bacterial phenotypes. Numerous candidate attachment proteins have been identified in this way, including P58, SARP, and the plasmid-borne protein P32 (**Table 2**) (Wayadande and Fletcher, 1998; Berg et al., 2001). Another very abundant membrane protein of *S. citri* that has been implicated in transmission is spiralin. *S. citri* mutants compromised in spiralin production exhibit reduced transmission by the vector, *Circulifer haematoceps* (**Table 2**) (Gasparich, 2010). This suggests that spiralin may mediate pathogen interactions within the insect vector, though specific mechanisms remain unknown.

### Phytoplasmas

Phytoplasmas are another category of the Mollicutes that depend on insect vectors for transmission (**Table 1**), but unlike *Spiroplasma*, they have pleomorphic shapes and are very difficult to culture (Contaldo et al., 2012). Phytoplasmas are a diverse monophyletic group, with more than 30 “*Candidatus* Phytoplasma” species described and 100s of subgroups (Hogenhout et al., 2008b). As a taxon they have a wide host range, infecting more than 800 different plant species (Hogenhout et al., 2008b), but individual strains have highly restricted insect and plant hosts. Collectively, more than 1000 plant diseases are caused by phytoplasmas that are transmitted by leafhoppers and, to a lesser extent, a few other hemipterans (Mitchel, 2004; Weintraub and Beanland, 2006).

Phytoplasmas have the smallest genomes of all described phytopathogenic bacteria, averaging ~0.7 Mb with a low G+C content, high number of repetitive regions, and interesting variability in genome features across the taxon (**Table 2**) (Kube et al., 2012, 2014). At least six types of ATP-binding cassette (ABC) transporters are conserved in the evaluated genomes. ABC transporters shuttle metabolites across bacterial membranes, and are predicted to allow nutrient and metabolite uptake from the host. Other common features include a superoxide dismutase enzyme (SOD), possibly used to counteract reactive oxygen species produced by hosts, and a protease (HflB), which is a virulence factor for ‘*Candidatus* Phytoplasma mali (Wang et al., 2014). Recently a conserved Mollicutes adhesion motif (MAM) was identified in the Onion Yellow Phytoplasma genome. This candidate protein (P38) interacts with crude insect extracts and weakly with plants extracts (**Table 2**) (Neriya et al., 2014), however, specific host targets are unknown.

Phytoplasmas also encode translocase *SecA*, part of the Type II secretion system for bacteria. This secretion system allows the delivery of functionally distinct proteins with a characteristic signal peptide at the *n*-terminal to the bacterial membrane. Because phytoplasmas have a single membrane, after the signal peptide is cleaved the proteins are released into the host environment (secreted). Secreted phytoplasma proteins can alter host functions and act as effectors (Bai et al., 2009). A single phytoplasma genome can encodes over 50 secreted proteins (SAP’s), however, the function of each one during host colonization and propagation is only known for a few (Bai et al., 2009). SAP effectors often alter host function by manipulating plant hormone homeostasis. For example, the effector TENGU inhibits auxin-related pathways leading to a dwarf plant phenotype and floral sterility (**Table 2**) (Minato et al., 2014). Further, *Arabidopsis* transgenic lines expressing SAP11, produce less jasmonic acid (JA) compared to controls (**Table 2**) (Sugio et al., 2014). This leads to abnormal vegetative growth and increased fecundity for leafhopper vectors on infected plants (Lu et al., 2014b). SAP effectors can also modulate pathogenicity through changes in development. SAP54/PHYL interacts with floral transcription factors and promotes degradation of the MADS-box proteins. MADS-box proteins are critical for floral meristem development and plants expressing SAP54/PHYL flower abnormally (**Table 1**) (MacLean et al., 2014; Maejima et al., 2014).

A second group of proteins delivered by the Sec-secretion system are the immunodominant membrane proteins (IMPs), which remain anchored and decorate the external membrane of phytoplasmas. IMPs are unique for phytoplasmas and are categorized into three subgroups depending on whether the n- or c- terminal side of the protein is exposed extracellularly (Amp, IdpA, or Imp; Kakizawa et al., 2006). When a monoclonal anti-AMP from “*Candidatus* Phytoplasma asteris” Chrysanthemums Yellows strain (CPY) was fed to the leafhopper vector, internalization of the phytoplasma and transmission efficiency was reduced. These results imply that anti-Amp impedes attachment of the bacteria in the vector gut (**Table 2**) (Rashidi et al., 2015).

### Liberibacter

The genus *Liberibacter* spp. contains six species of phloem-limited bacteria (Haapalainen, 2014): “*Ca*. Liberibacter africanus,” “*Ca*. Liberibacter americanus,” “*Ca*. Liberibacter asiaticus,” “*Ca.* Liberibacter solanacearum,” “*Ca*. Liberibacter europaeus,” and *Liberibacter crescens*. “*Ca*. Liberibacter africanus,” “*Ca*. Liberibacter americanus,” and “*Ca*. Liberibacter asiaticus” are associated with citrus greening disease, also referred as Huanglongbing (HLB) in different regions around the globe (Gottwald, 2010). “*Ca.* Liberibacter solanacearum” (=“*Ca*. Liberibacter psyllaurous”) is phytopathogenic to members of the Apiaceae and Solanaceae plant families. These four species all depend on psyllid vectors for transmission and as alternative hosts (Fagen et al., 2014b; Haapalainen, 2014). “*Ca*. Liberibacter europaeus” has also been associated with psyllids, but its role as a plant pathogen has not been demonstrated. To date, only *Liberibacter crescens* has been cultured *in vitro*, but it is not considered phytopathogenic and it is not vector-borne (Fagen et al., 2014a). *L. crescens* was first isolated as a bacterial endophyte from papaya and has not been re-isolated in nature. Non-psyllid hemipterans may also be able to pick up the bacteria during feeding as bacterial DNA has been found in mealybugs (Pitino et al., 2014), however, liberibacter transmission by other hemipterans is currently not clear.

Liberibacters have a small genome of ~1.2 Mb. Comparative genomics have shown a similar gene organization across the genus and evidence of horizontal gene transfers as prophages integrated into the genomes (**Table 1**) (Thompson et al., 2015). Similar to phytoplasmas, liberibacters lack biosynthesis genes for amino acids, sugars, and nitrogenated bases, which imply they obtain those metabolic products from their host (Thompson et al., 2015). Accordingly, many ABC transporters are encoded in liberibacter genomes (Lin et al., 2011; Mafra et al., 2013; Yan et al., 2013). Active importation of nutrients from phloem and insect vectors may lead to nutrient imbalances, partially explaining the foliar symptoms observed in liberibacter-infected plants (Rashed et al., 2013).

Potential pathogenicity mechanisms of liberibacters have recently been suggested based on comparative bioinformatics with other phloem-limited bacteria. Liberibacters encode the basic proteins for Sec-dependent translocation, similar to phytoplasmas (Lin et al., 2011; Yan et al., 2013). However, as liberibacters have two membranes of different composition in contrast to phytoplasmas, it is not known whether putative liberibacter Sec-transported proteins cross the outer membrane and interact with the plant or insect host. Recently, two unusual autotransporters were identified in the liberibacter genome (*LasAI* and *LasAII*) and these may serve as an alternative secretion system to the Sec-system (**Table 2**) (Hao et al., 2013). Evidence suggests that plant transcripts and metabolites related to salicylic acid (SA) production are altered during ‘*Candidatus* Liberibacter solanacerum’ infection (Casteel et al., 2012; Chin et al., 2014). SA is an important signaling molecule involved in plant defense to pathogens and insects (Glazebrook, 2005; Walling, 2009; Erb et al., 2012). Recently, a NahG-like salicylate hydroxylase gene was found in the liberibacter genome. NahG is predicted to cleave salicylates derived from SA (Lin et al., 2013) and may be used to modify the plant defense system. Although comparative bioinformatics has revealed many potential proteins used by liberibacter to alter plant and vector metabolism and vector–plants interactions, exact mechanisms for host colonization and transmission remain largely unknown.

## Approaches to Study Vector-Borne Bacteria

The current understanding of pathogenicity mechanisms in vector-borne bacteria is largely influenced by the ability to culture those bacteria. To date only *X. fastidiosa* and *Spiroplasma* spp. have been cultured *in vitro* and both require very specific conditions (Dourado et al., 2015; Renaudin et al., 2015). Because of this limitation, much of the biology and mechanisms of host colonization for phytoplasmas and liberibacters are still poorly understood (Bove and Garnier, 2003). Another challenge of working with phloem-limited vector-borne bacteria in particular is the non-homogenous distribution in the phloem tissue. This makes choosing sampling locations difficult and can result in false negatives during detection. Further, symptoms vary significantly across plant hosts and do not necessarily correlate with pathogen titer. Despite these difficulties, approaches combining genomics, bioinformatics, transcriptomics, and genetic manipulation have contributed to recent advances in the understanding of how these bacterial pathogens colonize their host environments.

### Whole Genome Sequencing and Bioinformatics of Vector-Borne Bacteria

The complete genome sequences for many strains of vector-borne bacteria have recently become available (**Table 1**). This has allowed scientists to study bacterial gene function within these systems without the need to culture the organism. Bioinformatics can be used to compare genome sequences with the annotated genomes of close relatives or analyze sequences using server-based algorithms to assign predicted functions to each coding region (Rutherford et al., 2000). Amino acid sequences can be further explored to identify conserved patterns and domains. In this way, proteins with low average similarity can be assigned to a predicted function (Yu et al., 2010; Caccia et al., 2013; Cortazar et al., 2015). Finally, functions of unknown proteins can even be predicted using dedicated algorithms that identify patterns associated with signal peptides, localization, cleavage sites, phosphorylation, and transmembrane domains (Yu et al., 2010).

A limitation of these various bioinformatics approaches is that all programs are trained using cultured organisms. For unculturable bacteria, many unique sequences with no homologs in cultured species exist, making comparisons and inferences difficult (Kube et al., 2008). Despite these limitations, bioinformatics have been used successfully to study gene function for many phytoplasma effectors. Bai et al. (2009) identified 56 Secreted Aster Yellows Proteins (SAPs) in the genome of ‘*Candidatus* Phytoplasma asteris’ strain Aster Yellows Witches’-broom (AY-WB). In this study, they utilized a pipeline to predict prokaryotic signal peptides recognized by Sec-translocases (SignalP v. 3.0) and then predicted transmembrane domains within this list to predict secretion (TMHMM v. 2.0; Bai et al., 2009). Finally, the list of 56 predicted effectors was examined for eukaryotic nuclear localization signals (predictNLS and pSORT) to select SAPs targeting plant nuclei for further investigation (Bai et al., 2009; Lu et al., 2014b).

### Transcriptomics of Vector-Borne Bacteria in Their Hosts

After potential pathogenicity factors are identified, functional validation is required. For unculturable bacteria, transcription and translation of targets can only be evaluated within their hosts (plant or insect). This means RNA and protein isolations must be done from infected host tissue. By some estimates, only 0.1% of total RNA extracted from infected herbaceous hosts represents the phytoplasma RNA. Others report only 0.02% of the mRNA from the woody host was associated with the phytoplasma genome (Abba et al., 2014). However, high throughput sequencing technologies have expanded the possibilities for studying pathogens inside their hosts. Now RNAseq can be used to quantify the complete RNA population in a sample (Westermann et al., 2012). This technique has advantages over microarrays and qRT-PCR because it affords higher sensitivity for monitoring gene expression levels, independence from examining only known sequences, and wider detection ranges (Westermann et al., 2012). However, for vector-borne bacterial pathogens, RNAseq approaches have thus far had low levels of success.

RNAseq has been used to examine “*Candidatus* Phytoplasma mali” transcription in tobacco (*Nicotiana occidentalis*; Siewert et al., 2014). Prior to preparation for sequencing, total RNA was treated with a plant ribosomal depletion kit to enrich the samples for bacterial RNA. Only 0.003% of the total reads (17,046,418 reads averaging 115 b) were mapped against the protein coding regions of the predicted “*Candidatus* Phytoplasma mali” genome. Mapped reads corresponded to 132 genes out of the 497 predicted genes. In another RNAseq study, RNA was enriched for bacterial transcripts using a ribosomal depletion kit to remove plant cytoplasmic, mitochondrial, and chloroplast ribosomal RNA (Abba et al., 2014). Despite the enrichment and relatively deep sequencing, only 0.01% of the total reads (125,813,174 and 129,412,231, for each library) were mapped to the draft genome of the phytoplasma flavescence dore (Abba et al., 2014). In two slightly more successful studies, total RNA from psyllid vectors was used to detect transcripts from “*Ca.* Liberibacter solanacearum” (Ibanez et al., 2014; Yao et al., 2016). However, only 0.3% of the total (70,869,948) reads were mapped to the bacteria genome after ribosomal depletion (Ibanez et al., 2014). Transcriptomics offer a unique opportunity to overcome the many difficulties posed by these difficult pathosystems, but as evident in the above examples, many technical challenges remain.

### Genetic Manipulation of Vector-Borne Bacterial Phytopathogens

The first approach that permitted gene function discovery for vector-borne bacterial plant pathogens was the use of transposon mutagenesis with spiroplasmas in the early 1990s (Fletcher et al., 1998; Mutaqin et al., 2011). In this approach, a transposon with a selective marker was integrated randomly into the chromosome of *S. citri*, and recombinant colonies were selected in media with antibiotics. When transformed colonies were tested in the host, the transposon was retained for a few days without antibiotic pressure. This technique was used to determine that disruption of a solute binding protein (gene sc76) reduced transmission in the leafhopper vector (Boutareaud et al., 2004). Since this first study, numerous research groups have generated collections of *S. citri* mutants using this technique (Foissac et al., 1997; Boutareaud et al., 2004; Mutaqin et al., 2011). Currently, *X. fastidiosa* and *Spiroplasma* spp. are the only vascular plant pathogens transmitted by hemipterans for which genetic transformation protocols and mutant libraries are currently available.

Genetic manipulation using surrogate culturable bacteria and heterologous gene expression in plants has been used to test gene function for other vector-borne bacteria (Jain et al., 2015; Renaudin et al., 2015). In a study using the flavescence dore phytoplasma, the surface protein, variable membrane protein A (VmpA*)*, was expressed under the control of the *S. citri tuf* promoter in a recombinant *S. citri* (**Table 2**) (Renaudin et al., 2015). The *tuf* promoter was chosen because the *tuf* gene is expressed at high levels in most bacteria (Kim et al., 2009). In this system the leafhopper, *Euscelidius variegatus*, serves as a vector for both the phytoplasma and *S. citri*. Thus gain of function studies could be conducted with the recombinant *S. citri* in both hosts. In the case of phytopathogenic “*Candidatus* Liberibacter asiaticus,” a peroxidase protein (SC2_gp095) has been expressed in the cultivable *L. crescens* as a surrogate (**Table 2**). However, biological inferences from this system may be restricted by the lack of host infection of *L. crescens* after culturing.

An alternative method for studying gene function is to overexpress bacterial candidate proteins in the plant host. Model plants such as *Arabidopsis thaliana* and *Nicotiana* spp. are routinely used to evaluate gene function for plant pathogens. Once the candidate gene is selected, the coding sequence is cloned into a suitable expression vector and transgenic plants can be generated. Several authors have utilized plant heterologous expression systems to investigate the function of phytoplasma SAPs (MacLean et al., 2011; Lu et al., 2014a; Yang et al., 2015). In these studies transgenic *A. thaliana* expressing individual phytoplasma SAPs were screened for symptom development and plant abnormalities (MacLean et al., 2011; Lu et al., 2014a; Yang et al., 2015). After a relevant phenotype was identified, plant gene expression changes and plant proteins interacting with the phytoplasma proteins were examined (**Table 2**). A limitation of this approach is that only profound disturbances caused by a single bacterial gene can be identified. In addition, model plants may not serve as natural hosts for all vectored-borne bacteria and relevance of findings may be limited to an artificial system.

## Application of Knowledge for ‘Next Generation’ Control Strategies

Controlling vector-borne pathogens is difficult. Chemical control of insect vectors is the most widely used method, but in most cases insecticidal applications are not sufficient to contain the spread of these pathogens and associated diseases. Furthermore, insect resistance and environmental regulations have limited the viability of long-term application of insecticides. Host plant resistance has been successful for several high value crops (Bisognin et al., 2008; Riaz et al., 2008), including grapevine tolerance to Pierce’s disease. In these plants, *X. fastidiosa* infection occurs, but titer remains low in the plant (Riaz et al., 2008). Due to the long time periods required to identify resistance and produce new varieties, this method may not always be a practical choice for the more aggressive and devastating outbreaks. Overall, as research on vector-borne bacteria continues to flourish, a focus on the ‘next generation’ of control strategies is needed.

One recent approach to block transmission of vector-borne bacteria used chemicals intended to saturate the pathogen-binding site in the insect or on the bacteria surface, so the insect picks up fewer pathogen cells (Killiny et al., 2012). In this study, vectors were fed artificial diet supplemented with *X. fastidiosa* cells and different potential transmission-blocking chemicals. Multiple lectins, carbohydrates, and antibodies were evaluated for potential transmission blocking characteristics. After feeding on the diet-bacteria mixture, insects were transferred to healthy plants to determine transmission efficiency with and without the different chemicals (Killiny et al., 2012). Diets containing certain lectins (wheat germ agglutinin and concanavalin A), *N*-acetyl glucosamine carbohydrates, and certain antibodies reduced the transmission efficiency under greenhouse conditions. The authors suggest that lectins probably compete with the bacteria for the binding sites inside the insect, while carbohydrate saturate *X. fastidiosa* adhesions on the cell surface (Killiny et al., 2012). The interference approach has also been explored in phytoplasmas using antibodies against the extracellular membrane protein Amp, with some success in the lab (Rashidi et al., 2015). Recently, phage-display libraries have been used to evaluate antibodies and protein–protein interactions inside the insect vector. In this approach, each phage contains a known peptide and the binding capacity of the peptide to an extracellular bacterial epitope is evaluated (Huang et al., 2012). The exact mechanisms mediating the ability of specific chemicals to block transmission is still unknown, and it is not clear how this technology could be used in large-scale application. How, for example, might a natural population of insects be exposed to the transmission-blocking chemical?

Some of the most extensive research efforts on ‘next generation’ control technologies for vector-borne bacteria have focused on the use of nucleic acids in gene drive systems (Sinkins and Gould, 2006), and with RNA interference strategies (Gordon and Waterhouse, 2007; Nandety et al., 2015). The first concept was explored initially in the field of medicine, and is based on the concept of ‘selfish DNA’. Selfish DNA is a naturally occurring phenomenon where certain genetic elements, such as transposable elements and others, spread in the genome of an organism and in the population by making additional copies of themselves. It has been suggested that selfish genetic elements could be used for control as a gene drive system that carries additional genes with anti-pathogen effects (Sinkins and Gould, 2006; Gantz and Jasinskiene, 2015). Populations of insects transformed with transposable elements or with a transgenic *Wolbachia* strain could be released into the environment, permitting the gene drive system and ‘gene of interest’ to spread in the population and block plant pathogen associations (Sinkins and Gould, 2006). Obvious concerns with this method are public acceptance of transgenic organisms, non-target impacts, and the costs of implantation.

Nucleic acids can also be utilized as a control method by inducing RNA interference (RNAi). RNAi has already been successfully exploited in plants to control viruses in commercial production (Tricoll et al., 1995; Gonsalves, 2006; Fuchs and Gonsalves, 2007; Scorza et al., 2013) and successful control of bacteria has been demonstrated (Escobar et al., 2001). RNAi can also be used to control insect species, altering insect reproduction, physiology, or survival (Gordon and Waterhouse, 2007; Wuriyanghan et al., 2011; Nandety et al., 2015). Direct injection, bait feeding, or transgenic host plants can be used to induce RNAi in insects. As direct injection is not practical for large scale control, and bait feeding is not effective in field studies for hemipteran insects, transgenic plants are the best options for using RNAi to control vectors of bacterial pathogens. While there is much excitement about the use of RNAi as an alternative control strategy (Gordon and Waterhouse, 2007; Donald et al., 2012; Li et al., 2013; Yu et al., 2016), additional research on delivery, safety, and non-target effects needs to be explored. Despite these unknowns, RNAi studies still represent an excellent attempt at next-generation control for these important plant pathogens.

## Conclusion and Future Directions

Devastating outbreaks of citrus greening disease, Pierce’s disease, and zebra chip disease in recent years have contributed to a rapid growth in the literature on bacterial plant pathogens and their hemipteran vectors (Haapalainen, 2014; Almeida and Nuney, 2015; Orlovskis et al., 2015). Whereas most plant-infecting viruses depend on hemipterans for transmission, most plant-infecting bacteria do not. The small subset of known bacteria that are vector-borne are able to propagate in both the plant host and the insect vector, organisms from diverse phylogenetic kingdoms (**Table 1**). This is in contrast to the non-propagative relationships most vector-borne plant viruses share with hemipteran vectors. The ability to transition between divergent hosts is remarkable considering that most vector-borne bacteria have highly reduced genomes compared their free-living ancestors, yet, we still do not understand the mechanisms which make this sort of transitioning possible. Variation in vector-pathogen specificity also exists across hemipteran groups (**Figure 1**; Supplementary Table S1), suggesting there are still unknown constraints on these relationships. Clear differences occur between the relationships that phloem and xylem colonizers share with insect vectors (**Table 1**). However, conclusions based on tissue tropisms should be made with caution, as only one xylem-limited vector-borne species has been identified so far.

The analysis of the genetic mechanisms mediating interactions between vector-borne bacteria and their hosts has focused largely on membrane-bound proteins and Sec-dependent peptides in gram-positive bacteria (**Table 2**). For gram-negative bacteria, the primary focus has been on toxins, enzymes, and aggregation factors (**Table 2**). Clearly, the role of membrane-associated proteins and extracellular structures represents the first target for investigating physical recognition inside the vector and initiation of host processes. However, the methodological bias toward these functional categories may limit our understanding of other important mechanisms mediating interactions with hosts. For example, in ‘*Candidatus* Phytoplasma mali’ 32% of the genome has no homology to any other sequences, and for ‘*Candidatus* Phytoplasma asteris’ strain Onion Yellow’s almost 50% of the genome is classified as unknown (Kube et al., 2008). Considering the highly reduced genomes and host-dependence of these bacteria, genes without an assigned function likely still play a significant role in the biology of the organism and will need to be investigated. The continued expansion of “omics” and other next-generation technologies in molecular biology will likely shed new light on the role of unknown coding sequences in host colonization, pathogenesis, and how host specificity may have evolved independently in different bacterial lineages.

Despite these advances, research on vector-borne pathogens is still in its infancy. Some of the most significant gaps in our understanding concern interactions with insect vectors. In particular, our understanding of leafhopper and psyllid feeding behavior, immunity, and plant responses to these insects needs to be improved. Genetic resources for these important vectors also need to be expanded. Promisingly, the genomes for the psyllid *Diaphorina citri*^1^ and at least one planthopper have been sequenced (Noda et al., 2008) and several other genome projects for important vectors of bacterial pathogens are underway (Evans et al., 2013; Poelchau et al., 2015). However, accessibility and quality control of insect genomic data remains an ongoing concern for the entomological community. In response to this, several projects attempting to address these issues have been initiated (Legeai et al., 2010; Poelchau et al., 2015; Yin et al., 2016), though at the time of publication most of these online resources remain works-in-progress. This area of research is likely to progress rapidly in the coming years. While climate change and the global food economy will continue to drive emergence of additional vector-borne bacterial pathosystems, the advent of genome editing, single-cell–omics, and interference RNA techniques will contribute to the identification of vector-borne bacterial phytopathogens and advances in our knowledge.

## Author Contributions

CC conceived the project. CC and LP-H wrote the article.

## Conflict of Interest Statement

The authors declare that the research was conducted in the absence of any commercial or financial relationships that could be construed as a potential conflict of interest.

## Footnotes

1 http://www.ncbi.nlm.nih.gov/genome/?term=txid121845[orgn]
