# Supplementary material for: Vector-Borne Bacterial Plant Pathogens: Interactions with Hemipteran Insects and Plants
Source: Front Plant Sci. 2016 Aug 9;7:1163. doi: 10.3389/fpls.2016.01163 (PMC4977473; doi:10.3389/fpls.2016.01163)
Supplement: Supplementary file 1 [file Table_1.DOCX]

**Supplemental Table 1.** Example of hemipteran suborders as vectors of virus or bacteria.

| HEMIPTERA VECTORS | | VECTOR-BORNE PATHOGEN | | |
| --- | --- | --- | --- | --- |
| SUBORDER STERNORRYNCHA | | **Type of plant pathogen** | **Transmitted organism** | **Reference/ Reviewed in** |
| Superfamily Psylloidea | *Diaphorina citri* Kuwayama | Bacteria | *“Ca.* Liberibacter asiaticus”  “*Ca.* Liberibacter americanum”  “*Ca.* Liberibacter africanus” | Haapalainen, 2014 |
|  | *Bactericera cockerelli* Sulc,  *Bactericera trigonica* Hodkinson*,*  *Trioza apicalis* Förster | Bacteria | *“Ca.* Liberibacter solanacearum” | Teresani et al., 2014 |
|  | *Cacopsylla picta* Forster  *Cacopsylla melanoneura* Forster | Bacteria | *“Ca.* Phytoplasma mali” | Seemuller and Schneider, 2004 |
|  | *Cacopsylla pyri* Linnaeus  *Cacopsylla pyricola* Forster | Bacteria | *“Ca.* Phytoplasma pyri” | Seemuller and Schneider, 2004 |
|  | *Cacopsylla pruni* Scopoli | Bacteria | *“Ca.* Phytoplasma  prunorum” | Seemuller and Schneider, 2004 |
| Superfamily Aleyrodoidea | *Trialeurodes vaporariorum* Westwood*,*  *Bemicia tabaci* Gennadius | Virus | *Begomovirus,*  *Crinivirus* | Gilbertson et al., 2015 |
| Superfamily Coccoidea | *Pheanacoccus* spp.,  *Pseudococcus longispinus* | Virus | *Ampelovirus* | Tsai et al., 2010 |
| Superfamily Aphidoidea | *Myzus persicae* Sulzer*,*  *Macrosiphum euphorbiae* Thomas | Virus | *Caulimovirus, Cucumovirus, Luteovirus, Potyvirus* | Hogenhout et al., 2008a |
| SUBORDER AUCHENORRYNCHA | | **Type of plant pathogen** | **Transmitted organism** | **Reference/ Reviewed in** |
| Superfamily Fulgoroidea | *Nilaparvata lugens* Stål | Virus | *Oryzavirus, Tenuivirus* | Hogenhout et al., 2008a |
|  | *Pentastiridius leporinus* Linnaeus | Bacteria | *“Ca.* Arsenophonus phytopathogenicus” | Bressan, 2014 |
|  | *Cixius wagneri* China | Bacteria | “*Ca.* Phlomobacter fragariae” | Danet et al., 2003 |
|  | *Cixius wagneri* China | Bacteria | *“Ca*. Phytoplasma sp.”  (Stolbur/16SrXII-A) | Weintraub and Beanland, 2006 |
|  | *Hyalesthes obsoletus* Signoret | Bacteria | *“Ca*. Phytoplasma sp.”  (Stolbur/16SrXII-A) |  |
|  | *Myndus crudus*van Duzee | Bacteria | *“Ca*. Phytoplasma sp.”  (Lethal yellowing/16SrIV) |  |
| Superfamily  Cicadoidea | *Diceroprocta*  *apache* | Bacteria | *Xylella fastidiosa* | Paião et al., 1996 |

| Supplemental Table 1. (Continued) | | | | |
| --- | --- | --- | --- | --- |
| HEMIPTERA VECTORS | | **VECTOR-BORNE PATHOGEN** | | |
| SUBORDER AUCHENORRYNCHA | | **Type of plant pathogen** | **Transmitted organism** | **Reference/ Reviewed in** |
| Superfamily Cercopiodea | *Philaenus spumarius* Linnaeus | Bacteria | *Xylella fastidiosa* | Saponari et al., 2014 |
| Superfamily Membracoidea | *Homalodisca vitripennis* Germar | Virus | *Phytoreovirus* | Whitfield et al., 2015) |
|  |  | Bacteria | *Xylella fastidiosa* | Chatterjee et al., 2008a |
|  | *Graphocephala atropunctata*  Signoret |  |  | Purcell, 2013 |
|  | *Macrosteles quadrilineatus* Forbes | Bacteria | “*Ca.* Phytoplasma spp.” | Purcell, 1982 |
|  | *Dalbulus maidis* Delong | Bacteria | *Spiroplasma kunkelii* | Ammar el et al., 2004 |
|  | *Circulifer tenellus* Baker | Virus | *Geminiviridae* | Soto and Gilbertson, 2003 |
|  |  | Bacteria | *Spiroplasma citri* | Wayadande and Fletcher, 1998 |
|  |  | Bacteria | *“Ca*. Phytoplasma sp.”  (Tomato big bud/16SrVI-A) | Shaw et al., 1993 |
|  | *Dalbulus maidis* Delong | Bacteria | *Spiroplasma kunkelii* | Ammar el et al., 2004 |
| SUBORDER HETEROPTERA | | **Type of plant pathogen** | **Transmitted organism** | **Reference/ Reviewed in** |
| Superfamily Lygaeoidea | *Piesma quadratum* | Virus | *Rhabdoviridae* | [Mitchel, 2004](#_ENREF_72) |
| Superfamily Coreoidea | *Anasa tristis* | Bacteria | *Serratia marcescens* | Bruton et al., 2003 |
| Superfamily Miroidea | *Stephanitis typica* Distant | Bacteria | *“Ca*. Phytoplasma sp.”  (Coconut Root wilt/ 16SrXI) | Weintraub and Beanland, 2006 |
| Superfamily Pentatomoidea | *Halyomorpha halys* Stâl | Bacteria | *“Ca*. Phytoplasma sp.”  (Paulownia witches'-broom/16SrI) | Hiruki, 1999 |
